# How Can High-Biodiversity Coffee Make It to the Mainstream Market? The Performativity of Voluntary Sustainability Standards and Outcomes for Coffee Diversification

**DOI:** 10.1007/s00267-016-0786-z

**Published:** 2016-11-12

**Authors:** Cecilia Solér, Cecilia Sandström, Hanna Skoog

**Affiliations:** School of Business, Economics and Law, University of Gothenburg, Box 600, Göteborg, 40530 Sweden

**Keywords:** Voluntary sustainability standards, Biodiversity, Marketing performativity, Coffee certification

## Abstract

This article investigates the outcomes of mainstream coffee voluntary sustainability standards for high-biodiversity coffee diversification. By viewing voluntary sustainability standards certifications as performative marketing tools, we address the question of how such certification schemes affect coffee value creation based on unique biodiversity conservation properties in coffee farming. To date, the voluntary sustainability standards literature has primarily approached biodiversity conservation in coffee farming in the context of financial remuneration to coffee farmers. The performative analysis of voluntary sustainability standards certification undertaken in this paper, in which such certifications are analyzed in terms of their effect on mutually reinforcing representational, normalizing and exchange practices, provides an understanding of coffee diversification potential as dependent on standard criteria and voluntary sustainability standards certification as branding tools. We draw on a case of high-biodiversity, shade-grown coffee-farming practice in Kodagu, South-West India, which represents one of the world’s biodiversity “hotspots”.

## Introduction

This article focuses on mass market voluntary sustainability standards (VSS) certifications as marketing tools for high-biodiversity coffee in the global coffee market. More specifically, it investigates how mass market coffee VSS certifications such as Rainforest Alliance (RA) and UTZ affect the ability to diversify based on biodiversity conservation and environmental conservation in coffee farming. VSS are “voluntary pre-defined rules, procedures and methods to systematically assess, measure, audit and/or communicate social and environmental behavior and/or performance of the firm” (Gilbert et al. 2011, p. 24). Third-party certification (TPC) is used to verify compliance with standards and labels on coffee to ensure standards compliance for the end consumer (Rasche 2015). The market for sustainable coffee has undergone a rapid transformation since 2008, with an annual average growth rate of certified or verified coffee production of 26 %, and VSS are recognized as strategic management tools for the coffee mass market and the specialty coffee market, which is increasingly moving into the mainstream (Potts et al. 2014). Today, coffee is the most standardized commodity on the global market; 40 % of all coffee produced was standards-compliant in 2012, compared with 15 % in 2008 (Potts et al. 2014).

Mainstream market VSS are marketing tools increasingly used by dominant actors in the coffee value chain to build trust among consumers who value sustainable-sourced coffee (Blackmore et al. 2012; Kolk 2013; Levy et al. 2016). These VSS are instrumental in coffee brand building among roasters and retailers, primarily in Europe and the United States (Blackmore et al. 2012; Kolk 2013; Levy et al. 2016). Mass market sustainability certification relies on sourcing of certified coffee from different coffee-producing countries to secure supply (Blackmore et al. 2012; Kolk 2013). Hence, for reasons of trust and recognition at the brand level, the sustainability assurance provided by these standards does not discriminate between high-biodiversity, shade-grown, and low-biodiversity sun-exposed coffee farming. Blending technology enables coffee roasters to mix and substitute coffees from different origins, qualities, and of different environmental and social standards as long as minimum criteria are met (Muradian and Pelupessy 2005; Varangis et al. 2003; Vellema et al. 2003). The need for large-scale sustainably sourced coffee for the mass market is proposed to generate a need to apply criteria that are sufficiently wide to allow for rapid certification in different coffee-producing contexts (Potts et al. 2014). Hence, coffees aimed at the mass market differ considerably from specialty coffees, which are valued based on geographic indications, quality, and taste (Muradian and Pelupessy 2005). However, through mainstream coffee actors such as Nespresso and Starbucks, the specialty coffee segment has increasingly moved into the mainstream, with specialty coffees representing 37 % of US coffee cups in 2012 (SCAA 2013).

VSS coffee certifications as marketing tools entail numerous benefits for coffee farmers, such as training opportunities, improved farming practice, product quality, and long-term trading relationships (Blackmore et al. 2012; Potts et al. 2014). The main driving force for growers to certify their coffee is the potential added value and economic viability of certification (Barham and Weber 2012; Chengappa et al. 2014), ensured by the large-scale demand for certified coffee in accordance with specific VSS. However, certification comes with a cost, and although considerable price premiums are required for farmers to carry certification costs successfully (Blackman and Naranjo 2012; Blackmore et al. 2012), the price premium on mainstream VSS-certified coffee varies considerably and is sometimes very low (Blackmore et al. 2012; Chengappa et al. 2014). For some farmers, certification is too costly in relation to the financial remuneration gained, and a few studies indicate that costs of certification affect biodiversity conservation (see Damodaran 2002; Neilson 2008a). Mainstream VSS are contested concerning their ability to provide environmental protection and their use to legitimize business activities (Gereffi et al. 2001; Raynolds et al. 2007; Hess 2008). When standards criteria are met or exceeded, as in the case of shade-grown coffee meeting minimum biodiversity criteria by default, incentives for coffee farmers to intensify environmental conservation efforts are reduced (Noblet and Teisl 2015). The VSS’ lack of discrimination between coffees farmed in alignment with standards and those exceeding these standards results in non-discrimination between high-biodiversity and low-biodiversity coffees; therefore, VSS can impede sustainable coffee production and consumption (Noblet and Teisl 2015).

From a marketing perspective, high-biodiversity, shade-grown coffee-farming practice is a valuable asset because the shade makes coffee unique and highly valued by the market. Shade-grown coffees are considered among the best in the world, particularly Indian and Ugandan robusta, which receive a high price premium on the world market (Karnataka Planters’ Association 2015; Ponte 2002). Coffee grown under shade occurs in complex agroforestry systems, and coffee estates play an important role in supporting the conservation of other habitats and biodiversity (Rao 2011; Bal et al. 2011; Chethana et al. 2010). The flavor of shade-grown coffee is considered superior to, and less bitter than, that of full-sun coffee (Upendranadh and Subbaiah 2012; Vaast et al. 2006). Hence, shade-grown coffee combines biodiversity conservation properties and unique qualities valued by the coffee market, which can improve farmer revenue (Vaast et al. 2006), and thus sustain high-biodiversity, shade-grown coffee farming. Speaking in terms of qualification of coffee, high-biodiversity coffee is singularized, or distinguished from competing coffees, based on taste characteristics (Callon et al. 2002). Given the high market value of shade-grown coffee and increasing mass market coffee VSS certification in regions where coffee is produced under shade (Kolk 2013), we must understand how such certifications affect coffee diversification, that is, the ability of high-biodiversity, shade-grown coffees to fully exploit the uniqueness of their properties in the global coffee market. We ask, “How do mainstream VSS affect high-biodiversity coffees potential to create value on the global market?”. We adopt a socially constructivist and performative approach in our study of the links between coffee diversification and VSS. From this perspective, VSS as marketing tools are not merely descriptive by making coffee-farming practices transparent but performative in the sense that the VSS “alter and remake social and material relations” (Konefal and Hatanaka 2011, p. 126), and thus intervene in the construction of markets (Araujo 2007; Muniesa et al. 2007). Marketing performativity studies focus on how concrete marketing activities affect markets (Mason et al. 2015). In this article, the outcomes of mainstream market coffee VSS are analyzed in terms of how they affect the marketing potential of high-biodiversity coffee on the global market. We use a model of market practice configuration (Kjellberg and Helgesson 2007) to show how mainstream coffee VSS participate in reinforcing established exchange practices in this market. We use the concept of market configurations to denote how market practices are configured to fit one another (Normann 2001; Storbacka and Nenonen 2011a). Our analysis provides an understanding of how VSS certifications configure the mainstream coffee market and affect the potential for coffee diversification based on biodiversity conservation.

The aim of this article is to describe how two mainstream VSS certifications, RA and UTZ, configure coffee mass market practice and generate outcomes relevant for the marketing of high-biodiversity, shade-grown coffee. We draw on a case of high-biodiversity, shade-grown coffee-farming practice in Kodagu, South-West India, which represents one of the world’s biodiversity “hotspots”. This region is a suitable case for three reasons: (1) The area of Kodagu is covered by rainforest and represents one of the world’s biodiversity “hotspots”. These hotspots are characterized by exceptional concentrations of endemic species (Myers et al. 2000), (2) Coffee from this region is highly valued by the world market (Karnataka Planters’ Association 2015; Ponte 2002), (3) Coffee growers in this region are increasingly certifying their estates according to UTZ and RA standards (Chengappa et al. 2014; Neilson and Pritchard 2007).

We contribute to marketing research that studies how issues of sustainability are defined and constructed by market actors and how these issues affect economic exchange (Boons and Mendoza 2010; D’Antone and Spencer 2014; Solér et al. 2015). In particular, we contribute to research on the performative power of VSS from a marketing perspective. The VSS literature approaches represent various perspectives (Ponte and Cheyns 2013). Institutionalist perspectives deal with private authority and the legitimacy of the organizations and stakeholders (Reinecke et al. 2012; Riisgaard 2009). The political economy perspective focuses on issues of environmental governance (Giovannucci and Ponte 2005; Ruben and Zuniga 2011). A marketing perspective on VSS highlights how markets are shaped by standards for marketing purposes and what environmental sustainability outcomes follow from dominating VSS in specific markets (D’Antone and Spencer 2014; Ponte 2012). In this article, we expand such an understanding. Based on our data we describe VSS as marketing devices that shape the mainstream coffee market through mutual reinforcing market practices as large-scale supply of low-priced sustainable coffee and low standards for biodiversity conservation in coffee farming.

## VSS Certifications as Marketing Tools

VSS certifications are valued as marketing and brand development tools in the coffee industry (Blackman and Naranjo 2012; Blackmore et al. 2012; Potts et al. 2014). UTZ is one of the largest mainstream coffee certification schemes, has partnered with Sara Lee, and has the largest share of sustainable coffee of all the large coffee roasters in the world (Kolk 2013). RA has developed a partnership with Kraft and Nespresso (Kolk 2013; Potts et al. 2014). For coffee roasters, the increasing mainstreaming of sustainable supply reflects the desire to maintain market position, to secure supply and to protect corporate reputations by investing in sustainable production practices (Bartley 2007; Ingenbleek et al. 2007; Neilson 2008b; Muradian and Pelupessy 2005). Investment in mainstream market VSS certifications also reflects an anticipated growth in sustainable consumer segments (Blackman and Naranjo 2012; Blackmore et al. 2012; Potts et al. 2014). It is assumed that consumers value and pay more for sustainably produced commodities and that this value is reflected in a demand for these commodities (Blackman and Naranjo 2012; Chiputwa et al. 2015; Giovannucci and Ponte 2005). Also the market mechanism is believed to work to improve producers’ environmental and social performance through price premiums and market access (Blackman and Naranjo 2012; Chiputwa et al. 2015; Giovannucci and Ponte 2005).

The need for large-scale sustainably sourced coffee for the mass market is proposed to generate an application of standard criteria that are sufficiently wide to allow for rapid certification in different coffee-producing contexts (Potts et al. 2014). The stringency of standards addressing the mainstream coffee market (e.g., RA and UTZ) differs from those VSS targeting coffee niche markets (e.g., Organic and Fairtrade). Standards vary in terms of criteria coverage and depth (Raynolds et al. 2007; Potts et al. 2014), and mainstream market VSS apply less stringent and less broad environmental and social certification criteria compared with niche market VSS (Potts et al. 2014). As an example, biodiversity-relevant criteria, such as shade-relevant control points in UTZ certification in terms of “adequate number per hectare of suitable shade trees”, and RA criteria, such as “the tree community on the cultivated land consists of a minimum of 12 native species per hectare on average”, are wide enough to cover a range of coffee management systems in terms of shade. In terms of biodiversity, broad certification criteria allow UTZ and RA to certify a range of different types of coffee production systems with varying biodiversity conservation properties. Critics view such broad criteria as applying “lowest common denominator global-scale coffee codes” (Neilson 2008b, p. 192); however, mass market VSS are viewed as the normative framework for mainstream coffee actors’ sustainability initiatives (Giovannucci and Ponte 2005; Neilson and Pritchard 2007). Niche market VSS such as Organic certification and FairTrade offer higher price premiums to farmers than RA and UTZ certifications do (Blackmore et al. 2012; Chengappa et al. 2014; Kolk 2013), which aim for the mainstream market by supporting increased coffee productivity as an incentive to coffee farmers (Levy et al. 2016).

VSS certifications as coffee-branding tools require standardized sign recognition that builds trust concerning sustainability claims among consumers of coffee (Blackman and Naranjo 2012; Potts et al. 2014). Mainstream VSS certify coffee from different regions globally, with inherent locally specific environmental challenges. However, as discussed in the VSS literature, there is a paradox in communicating environmental governance in agricultural production, which by default is localized, by the same sustainability metric across various coffee-farming localities (Tischner and Kjærnes 2010; Osmundsvåg 2010; Ponte and Riisgaard 2011; Giovannucci and Ponte 2005; Neilson and Pritchard 2007; Renard 2005). Broad biodiversity-related criteria, applied to coffee management systems in different coffee-producing regions each with localized environmental challenges, have implications for the ability of coffee consumers to distinguish between high-biodiversity and low-biodiversity coffees. Hence, such broad criteria affect high-biodiversity coffee’s potential to create value on the mainstream coffee market (Vaast et al. 2006).

## A Market Construction Perspective on VSS

From a market construction perspective, different representations of sustainable coffee, such as VSS with varying standard stringency, are translated into normative frameworks that correspond to specific exchange practices (D’Antone and Spencer 2014; Kjellberg and Helgesson 2007; Normann 2001; Storbacka and Nenonen 2011a). There is an assumption that market elements are configured to fit one another (Normann 2001; Storbacka and Nenonen 2011a). This assumption implies that, in the case of the marketing potential of high-biodiversity coffee, coffee mass market VSS certifications are intimately linked to market norms (related to biodiversity) and to specific financial remuneration and sourcing schemes (Boons and Mendoza 2010; D’Antone and Spencer 2014; Levy et al. 2016). As an example, VSS as marketing tools framing the mainstream coffee market attribute certain biodiversity qualities to coffee through criteria setting, while simultaneously treating other biodiversity-related issues (possibly those connected to high costs) in coffee farming as so-called externalities (Callon 1998; Callon et al. 2002; Neyland and Simakova 2010; Onyas and Ryan 2015b). To contain such externalities within the mainstream coffee market would require a requalification of biodiversity coffee and a subsequent change in sourcing and producing schemes.

**Fig. 1:**
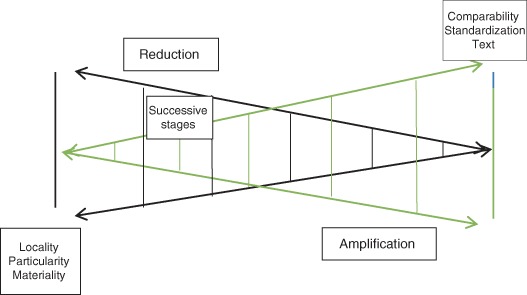
Trade-off between what is gained (amplification) and what is lost (reduction) in information production. *Source*: Latour (1999, Fig. 2.22, p. 71)

The performativity of VSS criteria is particularly useful for understanding how coffee diversification is affected by the use of VSS in coffee branding and marketing. Standards tend to become concrete and replace “reality” when used in certification schemes (Mason et al. 2015; Latour 1999). Figure 1, originally an illustration of how soil scientists categorizing a specific soil sample must classify and code a number of soil samples to do so, shows a trade-off between amplification and reduction in information production (Latour 1999). This suggested trade-off can help us clearly see the mechanisms of standardization in terms of loss of locality and particularity. In the case of high-biodiversity coffee, the locality and particularity of this coffee is the very base for its perceived value on the global coffee market, as the shade cover in high-biodiversity coffee farming creates the unique taste that is highly valued by the market.

**Fig. 2:**
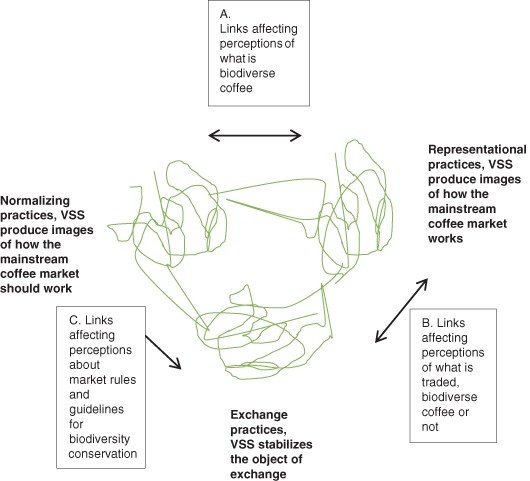
Links between how VSS contribute to exchange, representational and normalizing practices in the coffee market and affect perceptions of biodiverse coffee. This figure is an adaptation of Fig. 2 in Kjellberg and Helgesson (2007)

To comprehend the relationship between mass market VSS certification and the marketing value potential of high-biodiversity and shade-grown coffee, we describe the mainstream coffee market as a market configuration in which market elements are configured to reinforce one another and achieve a high degree of configurational fit between elements (Storbacka and Nenonen 2011a). The literature identifies two types of configurative elements: market practices, that is, the interaction between market actors in a market configuration, and market actors, who take part in market practices (Araujo et al. 2008; Storbacka and Nenonen 2011a). The market practice literature suggests that markets can be conceptualized as being constituted by exchange practices (activities making economic exchange possible), representational practices (activities producing images of markets, bridging distances in time and between market actors), and normalizing practices (activities establishing objectives for how a market should work according to certain market actors) (Kjellberg and Helgesson 2007). The three categories of market practices are not distinct but rather are best understood as “dense areas of activity” (Kjellberg and Helgesson 2007, p. 145). VSS certifications participate in the making of the mainstream coffee market because they affect economic exchanges occurring in the market through the images they represent of what “coffee is sustainable” (representational practices) and through the criteria and certification established by these standards (normalizing practices). Exchange, representational and normalizing practices are linked together through the process of translation. Translation refers to how something, in this case coffee VSS certification, is transformed into something else (Callon 1986). Figure 2 illustrates how coffee VSS contribute to linking exchange, representational and normalizing market practices so that these market practices seem to have a good fit because the practices reinforce one another through linkages between them (Storbacka and Nenonen 2011a). The figure serves the purpose of making the abstract notion of translation concrete in the case of VSS configuring coffee market practice. First, in a very simplified form, Fig. 2 presents the linkages between VSS as representing an image of what “coffee sustainably farmed according to specific standards” means, that is, representational practice, and these standards as objectives concerning how the sustainable coffee market should work, that is, normalizing practices. These linkages (link A) affect market perceptions of what biodiverse coffee is. Here representational practices (how biodiversity in coffee farming is described in specific sustainability standards) affect the agreement among coffee market actors regarding how biodiversity should be measured and attributed to coffee (normalizing practices). In a similar vein, normalizing practices (market actors’ agreements regarding VSS as a legitimate biodiversity indicator for coffee) impact what biodiversity criteria are part of VSS standards (representational practices). Second, Fig. 2 presents linkages between normalizing practices and VSS as stabilizing the exchanged coffee in terms of remuneration and supply schemes, that is, exchange practices, that affect whether and how VSS certification standards (in terms of biodiversity conservation) are translated into market rules and guidelines (link C). Hence, the stabilization and framing of biodiverse and sustainable coffee (and interlinked schemes of supply and remuneration) as VSS-certified and labeled coffee exchanged on the global market (exchange practices) influence the stability of norms (agreement among market actors) and the perceived legitimacy of VSS biodiversity criteria and certification (normalizing practices). These normalizing practices affect and serve the stabilization and framing of biodiverse coffee needed for coffee exchange to take place. Third, Fig. 2 illustrates linkages between representational practices such as images of biodiverse coffee according to VSS, and exchange practices that affect market perceptions concerning whether biodiverse coffee is traded or not (link B). In more concrete terms, representations of biodiverse coffee as VSS-certified and labeled coffee (representational practices) influence interaction and subsequent exchange between coffee buyers and sellers (exchange practices). As VSS (biodiversity) critera are applied in coffee exchange practices they sustain the image of the coffee market as dealing with issues of biodiversity in coffee farming (representational practices).

From this line of reasoning, it follows that VSS affect diverse coffee market activities and have configurational properties valid for the value of biodiverse coffee on the mainstream market. Hence, we can refine our initial question and ask “How do mainstream VSS certifications configure the mainstream market for high-biodiversity coffees?”.

Given our interest in the configurational properties of VSS valid for the value of high-biodiverse coffee on the mainstream coffee market, the market practice literature is informative on how possible reconfiguration of this market can be achieved that affect the value of such coffee (Storbacka and Nenonen 2011a, b). A market actor trying to script a given market, that is, change the configurational fit of market practice, is labeled a focal actor (Storbacka and Nenonen 2011a, b). Market scripting, as the “conscious activities conducted by a market actor to alter current market configuration in its favor” (Storbacka and Nenonen 2011a, b, p. 259), implies that VSS can play a crucial role as a focal actor in advancing the value position of high-biodiversity coffee within the coffee market through applying more stringent biodiversity criteria. The scripting strength of an actor is dependent on positions of relative power in the market in terms of access to resources, information, and relationships, and of skills (Fligstein 2001; Zaheer and Bell 2005). By applying more stringent biodiversity criteria, mainstream coffee market VSS would have an effect on normalizing practices as changed perceptions of how the coffee market works according to experiences of prior market transactions and logics would follow (Brooks 1995; Normann 1977; Storbacka and Nenonen 2011a, b). Such perceptions affect predominant market actors’ mental models regarding how to create value (Storbacka and Nenonen 2011a, b) and business models through elements of market offerings, such as price, technology, and network architecture, such as supplier remuneration schemes (Mason and Spring 2011).

## Methodology

To describe two mainstream VSS certifications, RA and UTZ, as configuring coffee mass market practice and generating outcomes relevant for the marketing of high-biodiversity, shade-grown coffee, we have conducted an exploratory single case study. This case study is based on a literature review of Indian coffee farming from a biodiversity perspective and interviews with actors in the coffee value chain in Kodagu, India, and with representatives of the Indian government, the research community, and the RA. Given the exploratory character of this research, an accurate and multidimensional image of mainstream VSS certifications in Indian coffee farming and marketing was particularly important (Eriksson and Kovalainen 2008). A single case study approach is appropriate when investigating an extreme and unique situation (Yin 2009).

As part of this case study, 22 semi-structured personal interviews were conducted during 4 weeks in India in March and September 2014, one interview was held in Copenhagen (with a researcher) and one interview was conducted via Skype from Sweden (with a representative of RA). The following actors were represented among the interviewees (see Table 1): coffee growers from Kodagu, large coffee exporters/traders with ownership of estates in Kodagu, representatives of the Coffee Board of India, local exporters/traders, researchers into forest conservation and private governance of global value chains, and one representative from the RA. Respondents were recruited through “snowball” and convenience sampling (Saunders et al. 2009). Our personal contact, a board member of the Karnataka Planters’ Association, established the initial contact with coffee growers in the area and, through a snowball effect, with other medium-sized coffee growers with either RA-certified or UTZ-certified coffee estates. Researchers, coffee exporters/traders, and representatives of the Coffee Board of India were successively approached. In addition, coffee exporters/traders trading with RA-certified or UTZ-certified coffee from Kodagu were approached through a formal e-mail to participate in the research.

**Table 1:** Research interview respondents

Respondent	Role in the Indian coffee value chain	Size of coffee estate	TPC	Comments
Representative from RFA	Certifying body			
Grower	Coffee estate owner	Medium	RFA	Group-certified
Grower	Coffee estate owner	Medium	UTZ	Group-certified
Grower	Coffee estate owner	Medium	–	Left the UTZ group certification program
Grower	Coffee estate owner	Medium	UTZ	Group-certified
Grower	Coffee estate owner	Medium	–	Left the UTZ group certification program
Grower	Coffee estate owner	Medium	UTZ	Group-certified
General manager	Coffee estate owner and trader/exporter	Large	RA, UTZ, Organic	Individually certified
General manager	Coffee estate owner and trader/exporter	Medium	Organic	Individually certified
General manager	Coffee estate owner and trader/exporter	Large	–	Actively opposes TPCs
Manager	Coffee estate owner and trader/exporter in MNC	Large	RA, UTZ, SA8000, Organic	Individually certified
General manager	Coffee estate owner and trader/exporter in MNC	Large	RA, UTZ, SA8000, Organic	Individually certified
General manager	Local coffee trader/exporter in MNC		UTZ	Certifies estates under group certifications
General manager	Local coffee trader/exporter in MNC		RA, UTZ	Certifies estates under group certifications
General manager	Local coffee trader/exporter in MNC		RA, UTZ	Certifies estates under group certifications
Country manager	Coffee trader/exporter in MNC		RA, UTZ	Certifies estates under group certifications
Head of R&D	Coffee trader/exporter in MNC with own plantations	Large	RA, UTZ	Individually certified
Researcher	College of Forestry			Research in the field of coffee cultivation and biodiversity conservation
Researcher	Copenhagen Business School			Research in the field of governance and TPCs
Representative from the Coffee Board of India	Government body			
Representative from the Coffee Board of India	Government body			
Researcher from the Coffee Board of India	Government body			

Interviews with growers and estate owners focused on how coffee-farming practice is influenced by UTZ and RA certification. Furthermore, questions were asked about the incentives to certify. Several visits and stays on coffee estates gave us a better understanding of Indian coffee-farming practice. Interviews with exporters/traders, researchers, and representatives of the Coffee Board of India were aimed at further elucidating the incentives and effects on the level of certification for a coffee plantation. The interviews differed in length from 30 to 120 min and were tape-recorded and transcribed. In some cases, written notes were taken on site. The empirical findings were analyzed in a manner suggested by Eriksson and Kovalainen (2008), called the “case record” method. This approach is appropriate for the development of an accurate case description in cases with many unedited empirical data items from several sources. We were interested in developing themes from the empirical data, and theoretical concepts were used to structure the data (Andersen and Kragh 2011). The method of cross-checking, or triangulating, data from multiple sources helped to distinguish different behaviors and activities and to organize themes (Eriksson and Kovalainen 2008).

We started the analysis of our transcribed interviews by reading the interviews one by one to gain an overall understanding of the subjects discussed. The coding of transcripts using themes from literature and from interviews, and the extrapolation of cross-interview patterns, formed an iterative analytical process. The emerging themes were tested across interviews in order to ensure a critical analysis of the themes’ reliability (Eriksson and Kovalainen 2008) and rigor in the case study process (Yin 2003).

## The Case

### The Context—Kodagu Coffee on the World Market

**Fig. 3:**
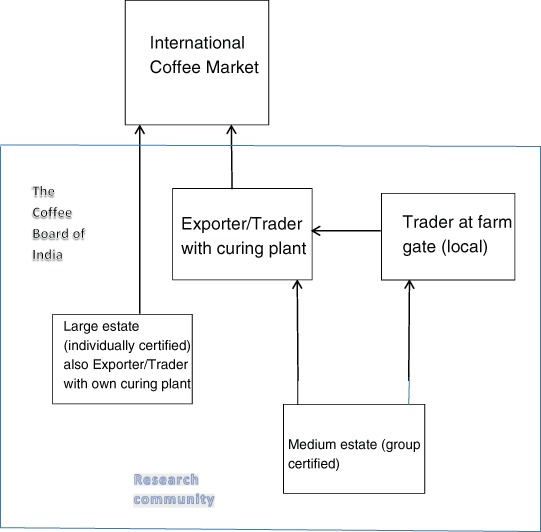
The international coffee value chain in Kodagu

India produces 3.6 % of the world’s coffee (International Coffee Organization (ICO) 2014). One-third of the production comes from the coffee-producing region of Kodagu, where coffee has been grown for the past 120 years. In 2014–2015, approximately 85 % of Indian coffee was exported (The Coffee Board of India 2016). The international Kodagu coffee value chain (see Fig. 3) includes large coffee estates with their own curing plants and medium-sized estates without their own curing plant. Large estates sell their coffee directly to roasters or branded companies in the international market. Medium-sized estates sell their coffee either to the local market or to an exporter/trader who takes the coffee to the international market. The Coffee Board of India, which is a governmental body under the control of India’s Ministry of Commerce and Industry, plays an important role in supporting the coffee industry and represents various interests for coffee growers, exporters/traders, curing plants, the labor market, and consumers (The Coffee Board of India 2014). The research community is actively involved in safeguarding the unique biodiversity features of the Kodagu region. As indicated in Fig. 3, the influence of the Coffee Board of India as well as the research community is limited to coffee trade within India.

#### Environmental benefits of shade-grown coffee farming in Kodagu

The area of Kodagu is covered by rainforest and represents one of the world’s biodiversity “hotspots”. These hotspots are characterized by exceptional concentrations of endemic species (Myers et al. 2000). Kodagu is famous for its tradition of shade-grown coffee. Because of the forestation cover, coffee cultivation occurs in complex agroforestry systems, and coffee estates play an important role in supporting the conservation of other habitats and biodiversity in the region (Rao 2011; Neilson and Pritchard 2007; Bal et al. 2011; Chethana et al. 2010). Coffee estates in Kodagu have a tradition of retaining native trees and intercropping coffee with other species, such as pepper, cardamom, areca, citrus, and commercial timber (e.g., silver oak), to improve the economic viability of the estates. The leaves improve the fertility of the soil, and the shade cover helps control pests and diseases, and lessens the need for irrigation. Specific land tenure, tree rights, and sacred groves, traditionally known as devarakadu, constrain growers from felling trees (Bhagwat et al. 2005a). Together with the coffee plantations, the devarakadu and the natural forests constitute a wildlife corridor, providing a contiguous habitat for tigers, elephants, leopards, and deer (Bhagwat et al. 2005a).

In India, as elsewhere, increased global competition in the coffee market has been met by measures to increase productivity. Measures for increasing coffee yield and income in the short term in the region of Kodagu include the opening of shade and replacing shade-loving Arabica coffee plants with Robusta plantations (with subsequent loss of biodiversity and an increasing need for inputs such as water, fertilizers, and pesticides) (Abraham et al. 2013; Chethana et al. 2010; CAFNET (Coffee Argo-Foresty Network) 2011; Neilson and Pritchard 2007). Another common means of increasing coffee-farmer income is to replace native trees with the commercial—and more profitable—silver oak. Silver oaks are fast-growing trees that serve as an alternative income source in a time of coffee price recession. This replacement affects the habitat value of coffee estates (Damodaran 2002; Garcia et al. 2010; Neilson 2008a) because falling leaves and branches of silver oaks take a longer time to decompose compared with those of the native trees. One-third of the forest cover in Kodagu has been lost in the last three decades, and the area under coffee cultivation has doubled, which has put pressure on the ecosystem (CAFNET (Coffee Argo-Foresty Network) 2011; Chethana et al. 2010).

#### Certification of coffee farming in Kodagu—processes, cost, and price premiums

To reassure roasters, retailers, and consumers within the global coffee market that Indian growers’ farming practices are sustainable, actors downstream of the Indian coffee value chain (traders and exporters) need Indian coffee to be certified (Ingenbleek et al. 2007; Renard 2005). In the Indian coffee-producing region of Kodagu, two major mainstream market VSS are present, namely, UTZ and RA, and coffee growers are increasingly certifying their farms to gain market access (Chengappa et al. 2014; Neilson and Pritchard 2007).

The certification process follows a similar sequence of steps independent of the governance body through which it is processed. The standards are developed by the standards-setting body Sustainable Agriculture Network (SAN) for the RA and by UTZ Kapeh for UTZ. Interested parties submit an application, and third parties then audit and monitor compliance (Chengappa et al. 2014). Coffee plantations can be individually certified or certified as a group of enterprises in the case of a large number of smaller holders (Kleemann et al. 2014). Certification costs are proportionate to plantation size but are lower for group certifications (Chengappa et al. 2014). Individual certifications are primarily undertaken by larger plantations (see also Tovar et al. 2005; Chengappa et al. 2014), who work directly with certifying bodies. Medium-sized growers are often certified under a so-called “group certification” (see also Tovar et al. 2005; Chengappa et al. 2014) initiated by an exporter/trader, which simplifies the certifying process and reduces the costs involved by certifying several growers at the same time. In Kodagu, local representatives for traders and exporters actively recruit and group-certify coffee growers. In many cases, coffee farmers are double certified, that is, the coffee grown on a specific farm carries both RA and UTZ certification. Local representatives for traders and exporters carry the cost of the group certification process; however, growers must carry the cost for additional investments in storage, protective clothing for workers, and documentation/book-keeping, all of which are required for UTZ and RA certifications (Neilson 2008b).

Price premiums for Indian mass market-certified coffee depend on the fluctuations in world coffee prices and the quality of the coffee (Upendranadh and Subbaiah 2012). For RA-certified and UTZ-certified Indian coffee, price premiums are low (US1c to US14c per pound weight of green) due to high coffee prices, and fewer planters are being reported as undertaking certification (Chengappa et al. 2014). Premium prices are only paid if certified coffee is sold to the company that has group-certified the plantation where the coffee is grown. Less than half of certified Indian coffee from Kodagu is actually sold as such, due to local traders buying coffee as non-certified at farm gates. This is a convenient alternative to transporting the coffee to the company holding the group certificate, which is often located at a considerable distance from the planter (Chengappa et al. 2014).

#### Coffee VSS criteria related to biodiversity conservation

The two mainstream VSS engaged in certifying coffee farms in Kodagu, UTZ and RA (Chengappa et al. 2014; Neilson and Pritchard 2007), contain biodiversity-relevant criteria that have a direct effect on biodiversity conservation through tree density (shade) and tree diversity in coffee farms. Table 2 presents a selection of criteria (for RA certification) or control points (for UTZ certification) that are related to biodiversity conservation in Indian coffee farms.

**Table 2:** RA and UTZ standard biodiversity-relevant criteria

VSS	RA	UTZ-Certified
	Criteria relevant for biodiversity conservation in coffee farming set by SAN’s Sustainable Agriculture Standards	Control points relevant for biodiversity conservation
	2.8 Farms with agroforestry crops located in areas where the original natural vegetative cover is forest must establish and maintain a permanent agroforestry system distributed homogeneously throughout the plantations. The agroforestry system’s structure must meet the following requirements:	CF B_1_: An adequate number per hectare of suitable shade trees are planted and/or maintained on coffee plots
	a. The tree community on the cultivated land consists of a minimum of 12 native species per hectare on average	G.B._47_: Organic fertilizers and by-products available at farm level are used initially and supplemented by inorganic fertilizer if nutrients remain lacking
	b. The tree canopy comprises at least two strata or stories	
	c. The overall canopy density on the cultivated land is at least 40 %	G.D. _112_: Threatened and endangered species in the production area are identified, communicated to group members, and protected
	Farms in areas where the original natural vegetation is not forest—such as grasslands, savannas, scrublands, or shrublands—must dedicate at least 30 % of the farm area for conservation or recovery of the area’s typical ecosystems. These farms must implement a plan to establish or recover natural vegetation within ten years	
	8.1 The farm must have an integrated pest-management program based on ecological principles for the control of harmful pests (insects, plants, animals, and microbes). The program must give priority to the use of physical, mechanical, cultural, and biological control methods, and the least possible use of agrochemicals	G.D. _113_: The group promotes ecological diversity by protecting and enhancing habitats and ecosystems

*Source*: SAN (2010) and UTZ Certified (2015)

**Fig. 4:**
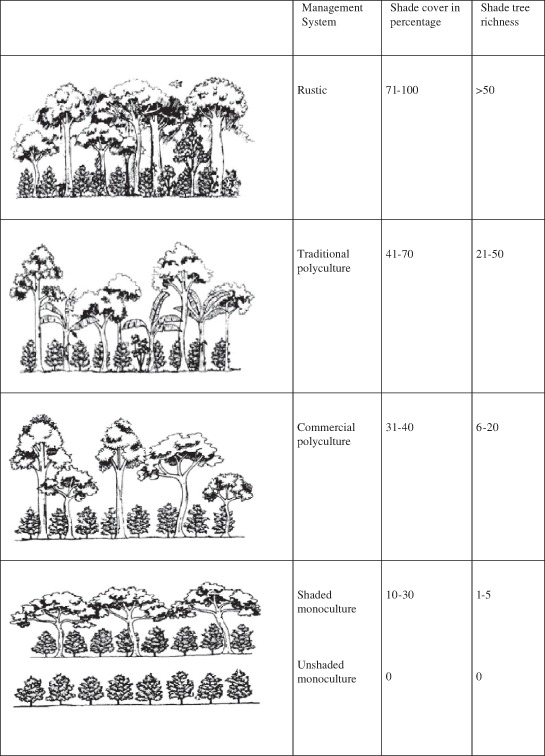
Diagram of the different coffee management systems, with approximate ranges in percent of shade cover and of shade tree species richness (Perfecto et al. 2005)

Table 2 shows that shade-relevant control points in RA and UTZ certification are open for interpretation and will harbor a wide range of coffee production systems under shade. UTZ states that biodiversity is conserved in coffee farms with an “adequate number per hectare of suitable shade trees”. RA criteria state that coffee farms must ensure that “ the tree community on the cultivated land consists of a minimum of 12 native species per hectare on average”, “the tree canopy comprises at least two strata or stories”, “the overall canopy density on the cultivated land is at least 40 %”, and “in areas where the original natural vegetation is not forest—such as grasslands, savannas, scrublands, or shrublands—must dedicate at least 30 % of the farm area for conservation or recovery of the area’s typical ecosystems”. Figure 4 presents the relationship between different coffee management systems in terms of shade cover (in percent) and shade species richness (Perfecto et al. 2005). When contrasting RA criteria for shade cover and tree richness in Table 2 (we here concentrate on RA because UTZ criteria on issues of shade are very broad) with the coffee management systems in Fig. 4, we can conclude that rustic, traditional, and commercial polyculture and shaded monoculture fulfill the RA criteria for biodiversity conservation. Shade percentage criteria encompass all four management systems depending on whether the natural vegetation is interpreted as forest or not. Criteria such as “the overall canopy density on the cultivated land is at least 40 %” and “in areas where the original natural vegetation is not forest—such as grasslands, savannas, scrublands, or shrublands—must dedicate at least 30 % of the farm area for conservation or recovery of the area’s typical ecosystems” imply that if forest recovery is ensured, shaded monoculture fulfills the shade percentage criteria. The shade diversity criterion that applies to agroforestry coffee farming, “the tree community on the cultivated land consists of a minimum of 12 native species per hectare on average”, is fulfilled by rustic, traditional, and commercial polyculture. This criterion can also be met by non-forest-shaded monoculture (grasslands, savannas, scrublands, or shrublands) if many native species are found in part(s) of the plantation that increase the number of native trees on average. Put in other words, non-forest-shaded monoculture (see Table 1), where 30 % of the farm is dedicated to recovery of the area’s typical eco-system and where recovery increases the average number of native species per hectare to a minimum of 12, lives up to the shade diversity criteria. The different regimes of shade in coffee farming (Perfecto et al. 2005) are presented in Fig. 4, all have different implications for biodiversity; however, all but one seemingly fit into the biodiversity criteria specified in the RA and UTZ standard for certified coffee. In Kodagu, shade intensity in coffee farming is categorized as high shade (canopy cover >70 %) with 94 % native trees on average, and low shade (canopy cover <70 %) with 80 % native trees on avergage (Chethana et al. 2010). Hence, Kodagu coffees encompass all shaded coffee management systems in Fig. 4 and fulfill RA biodiversity criteria regardless of shade intensity.

### Kodagu Coffee Actors’ Experiences with Mainstream Market VSS

In our analysis, three themes have emerged that relate to the experiences of actors in the Kodagu coffee value chain concerning mainstream market VSS and biodiversity conservation. The three themes—which are partly interrelated—are summarized in Table 3 and described further below.

**Table 3:** Kodagu coffee actors’ experiences of mainstream market VSS

Kodagu coffee actors’ experiences of mainstream market VSS
1. **Buyer-dominated definition of environmental protection**
VSS perceived as imposed on local actors in value chain
Group certification of smallholders initiated by exporters/traders
Buyer-driven sustainability standards
2. **Certification in the long-term buyer–farmer relationship**
Preferred supplier
Cost of certification
3. **No remuneration for environmental protection beyond standards**
Easy certification process
Inadequate price premium

#### Buyer-dominated definition of biodiversity conservation

One pertinent theme across interviews was that the criteria for biodiversity conservation in mainstream coffee certification standards are perceived as being defined by actors outside India. Growers feel that the formulation and enforcement of TPC standards are imposed on them.

They [the third-party certifier] had already decided what the conditions were [in Kodagu]. They looked at us suspiciously. They do not take the farmer as a stakeholder; they think the farmer is the bad guy.

(Grower, medium-sized coffee estate owner certified with RA)

These experiences are supported by Chengappa et al. (2014), who state that growers in Kodagu believe that certifications do not consider the local context and realities of Kodagu. Coffee growers in Kodagu have a long tradition of management practices that support the conservation of biodiversity in the region. The reasons behind this include the historical limited access to water for irrigation, in which shade has been essential to conserve water and protect plants from direct sunlight and draught.

I am the fourth generation of coffee farming, so they [the coffee growers] have been here for 200 years, and they have been looking after the environment—they have saved it.

(Grower, medium-sized coffee estate owner certified with UTZ)

Interviews with managers in Indian coffee trading clearly suggest that buyers on the international coffee market stipulate which VSS are to be implemented among coffee growers in Kodagu. The exporter/trader recruits growers to the VSS certification schemes that are in demand by international buyers by paying the initial costs and the annual fees for holding the certification.

As of now, the answer is no [to certify growers as organic]. It is all market driven. If someone asks us to go into organic farming, we would definitely do so. But if you cannot market it, it is not viable. And also, we are in the mainstream market if we focus on this market—it [organic farming] is not a business case.

(General Manager from an international coffee trader/exporter certified with UTZ)

Exporters/traders choose not to support stricter certification schemes—for example, organic coffee farming—because there is little demand for such coffee from downstream market actors. In this case, buyer-dominated definition of biodiversity conservation means that the international buyer is the primary driver of how biodiverse coffee farming is standardized. Coffee growers are dependent on the economic support given by exporters/traders to certify their coffee as part of securing coffee exchange in the long term. Some coffee growers actively oppose buyer-dominated definition of environmental protection in Kodagu. Mainstream market coffee VSS are perceived as not being able to distinguish the unique environmental features of Indian coffee farming. Without such discriminatory power, VSS are primarily viewed as a cost, not as an opportunity to capitalize on the uniqueness of high-biodiversity and shade-grown coffee.

#### Certification in the long-term buyer–farmer relationship

There are clear advantages for both coffee farmers and coffee exporters/traders in certifying Kodagu coffee, according to mainstream VSS. By certifying coffee growers, exporters/traders can establish closer bonds with growers and secure their sustainable coffee supply. Certification enables farmers to sell their coffee at a specified price, the so-called “price premium”. It should be noted that coffee growers are not required to sell their coffee to the particular exporter/trader; nonetheless, to get their coffee certified and to receive the expected price premium, the grower is required to sell to that particular company (Chengappa et al. 2014). Holding a certification represents a means for growers to become a “preferred supplier”. In the aftermath of the coffee crisis in the years 2000–2004, with declining prices and oversupply, the possibility of being able to sell coffee as certified to exporters/traders at a known price is perceived by many farmers as a means to secure long-term market opportunity.

During our study, several growers stated that mainstream VSS certification represents a necessary evil to uphold relationships and access to buyers and expressed the feeling of being “forced” into the program.

We are forced to have a certification. It is not driven by us.

(A large coffee estate owner certified with UTZ, with RA, and for organic production)

In light of increasing world demand for certified coffee, our results indicate clearly that the certification process is enforced by downstream actors in the international coffee chain, such as branded companies and roasters, who put pressure on upstream buyers and exporters/traders with operations in India to certify coffee growers in the area. The representatives of coffee exporters/traders, in both local and national management, draw a picture of ongoing and active recruitment of coffee farmers into both UTZ and RA certification programs due to the demand for certified coffee in the global coffee market. According to representatives of the Coffee Board of India, non-participation in VSS certification schemes represents a barrier to trade because growers increasingly have no option but to follow the growing demand from exporters/traders for certified coffee according to dominant VSS in the mainstream market (see also Kleeman et al. 2014).

The cost associated with certification—particularly multiple certifications—creates a need for increased income in Indian coffee farming.

#### No remuneration for environmental protection beyond standards

In line with the high biodiversity of shade-grow coffee cultivation in Kodagu compared with other coffee-producing localities in the world, coffee growers from the region who were interviewed expressed the ease of becoming certified (see also Chengappa et al. 2014).

It is a perfect fit [between criteria of VSS and nature]. You don’t have to do many other things, because it is a natural fit. You just need to do some brief things to maintain the certification. So, that is an advantage to get the certification.

(General Manager, large coffee estate and exporter/trader certified with RA, with UTZ, and for organic production)

The context-specific conditions of coffee farming in Kodagu are more or less biodiverse by default, and coffee growers easily fulfill the biodiversity requirements of RA and UTZ. The shaded coffee estates of Kodagu exceed the requirements concerning tree density and native species set by VSS (SAN 2010), and biodiversity conservation is not easily compared with that of sun-exposed coffee farming in Vietnam, Brazil, or elsewhere (Neilson 2008b). Certification implies only minor improvements to farming practice on the Kodagu estates. The most common changes forced by certification include buying safety equipment for laborers (such as gloves), small items of maintenance work on the estates, a restriction on the use and storage of fertilizers and pesticides, and administrative work such as book-keeping.

From a farmer’s perspective, the ease of becoming certified should be accompanied by a price premium.

By and large, I was already doing what was required [prior to getting certified]. All of it, and more! What was mandatory we already do. We even [provide] loans to the workers for marriage. No child labor, storage of chemicals, plastics disposals—all this was being done. This was why I was tempted to this [VSS certification]. So if I was going to get a premium for it? Why not? But I fight over the premium. The price I get from them [exporter/trader] is a discounted price, lower than the local market.

(Grower, medium-sized coffee estate owner certified with UTZ)

UTZ and RA certifications are two mass market VSS with prices negotiated between the buyer and seller. They do not guarantee an assured premium, but instead their philosophy is that quality improvements in production and processes will help realize market-determined quality premium and productivity gains (Kolk 2013; Levy et al. 2016). Consequently, many growers have expressed a feeling of being “fooled by the system”, and several of the interviewed growers have chosen to leave, or are considering leaving, the VSS certification schemes.

Indirectly I probably pay for the certification by getting a bad price.

(Grower, medium-sized coffee estate owner certified with UTZ)

## Analysis—How RA and UTZ Certifications Configure the Mainstream Market for High-Biodiversity Coffee

**Fig. 5:**
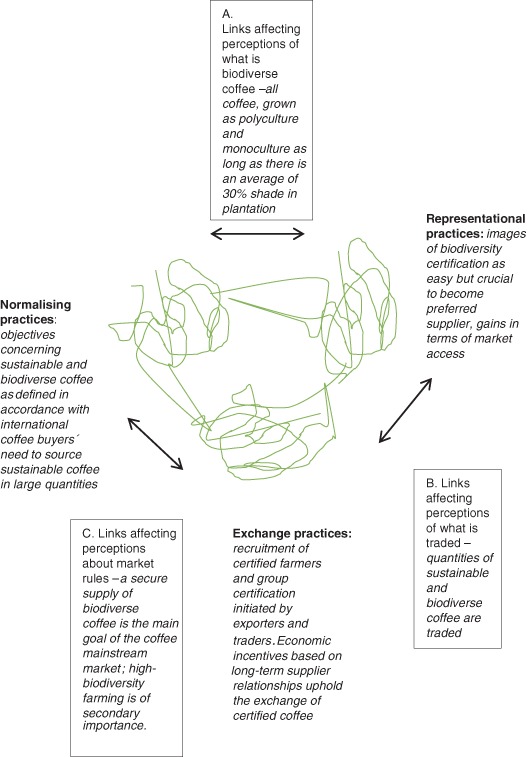
Exchange, representational and normalizing practices in the mainstream coffee market affected by RA and UTZ coffee certification, with outcomes for the marketing of high-biodiversity, shade-grown coffee. This figure is an adaptation of Fig. 2 in Kjellberg and Helgesson (2007)

Our findings provide input to an analysis of how the RA and UTZ certifications affect coffee mass market practices with outcomes relevant for high-biodiversity coffees’ potential to create value in this market. The three themes that emerged from the data on the experiences of actors in the Kodagu coffee value chain have been categorized as activities making economic exchange possible (exchange practices), activities producing images of markets (representational practices), and activities establishing objectives for how a market should work according to certain market actors (normalizing practices) (Kjellberg and Helgesson 2007). Despite the great numbers of activities influencing the configuration of the market for high-biodiversity coffee, a clear contribution to the configurational fit between mainstream coffee market practices is related to experiences of RA and UTZ certification. These experiences contribute to the configuration of the market for high-biodiversity coffee in a way that affects the value creation of high-biodiversity coffee (Fig. 5). The theme “Buyer-dominated definition of biodiversity conservation” provides evidence that RA and UTZ certifications contribute to perceptions that sustainable and biodiverse coffee is (and should be) defined in accordance with international coffee buyers’ sourcing of sustainable coffee in large quantities (normalizing practices in Fig. 5). RA and UTZ certifications are experienced being imposed on local farmers/traders/exporters in Kodagu and local exporters recruit and group-certify coffee farmers as RA or UTZ-Certified farms. RA and UTZ are the certification schemes in demand by international coffee buyers and these buyers set the rules for sourcing of sustainable and biodiverse coffee aimed for the mainstream market. Thus, certification of Kodagu coffee farms is required in order for Kodagu coffee to be part of the mainstream coffee supplier base (exchange practices in Fig. 5).

The theme “Certification in the long-term buyer–farmer relationship” provides evidence that being certified according to RA and UTZ schemes is crucial for long-term business relationship between coffee farmers and mainstream market exporters. Equally, it is clear that Kodagu coffee farmers are willing to pay certification costs (initial group certification costs are paid by the local exporter, administrative, equipment, and storage-related cost at the farm level are carried by the farmer), costs that are related to being a preferred supplier and part of the mainstream market supplier base. Certification thus provides security for coffee farmers as preferred suppliers and upholds the exchange of coffee aimed for the mainstream coffee market (exchange practices in Fig. 5) as well as provide an image of what it means for local farmers and exporters to be part of this market (representational practices in Fig. 5).

The theme “No remuneration for environmental protection beyond standards” provides evidence that RA and UTZ certifications contribute to perceptions of biodiversity conservation in coffee farming as an easily accessible dimension of being part of the global supplier base providing sustainable coffee to the *international mass* market. The ease of being certified is accompanied by (perhaps logically so) low price premiums on certified coffee. The price premiums on certified coffee do not cover the cost of certification and are perceived as inadequate by Kodagu coffee farmers. The economic gain of certifying one’s coffee farm is not related to price premiums. Rather coffee framers engage in exchange of certified coffee based on the perceived security of a long-term supplier relationship (exchange practices in Fig. 5), perceptions that sustain an image of certification as an easy but crucial undertaking (representational practices in Fig. 5).

The contribution of RA and UTZ certifications to the configurational fit between market practices in the mainstream coffee market, with outcomes for the marketing of high-biodiversity, shade-grown coffee, is understood as the links in Fig. 5. Links between representational and normalizing practices (link A) in Fig. 5 enable us to see how mainstream market images of biodiverse coffee such as RA and UTZ certified coffee, are translated into normalized and taken for granted market perceptions of how this market works in terms of biodiversity conservation and vice versa (how taken for granted perceptions of how this market works translate into representation of the market). These links work to sustain a configurational fit between mainstream coffee market practices through mutual reinforcement (Storbacka and Nenonen 2011a). On the one hand, RA and UTZ biodiversity standards provide stability regarding how biodiversity should be measured and attributed to coffee farming, that is, as coffee grown in polyculture and monoculture plantations with a minimum average of 30 % shade (link A direction representational to normalizing practices). On the other hand, such standards, once accepted, established and used as a legitimate biodiversity indicator for coffee sustained by certification and labeling procedures, biodiversity standards seem to reflect a consensus regarding what it means that coffee is produced in a manner that ensures biodiversity conservation (link A direction normalizing to representational practices).

The biodiversity standards play an important role in establishing a fit between normalizing and representational practices in the mainstream coffee market. Figure 1 (Latour 1999) illustrates a process in which localized and particular biodiversity in the real world of coffee farming is reduced to allow VSS criteria to define biodiversity in a standardized and compatible manner across coffee farming in different regions and using different management systems (this is called reduction in Fig. 1). When VSS biodiversity criteria (see Table 2) are applied to localized coffee farms, they are amplified in scope (this is called amplification in Fig. 1). Hence, every effort to describe reality, in this case biodiversity in coffee farming, transforms our understanding of this reality, and biodiversity criteria in coffee VSS certification seem real when applied (Latour 1999). In practice, standard reduction in locality and parallel increasing compatibility serve to sustain, as criteria are widely applied, perceptions of how biodiversity should be measured and attributed to coffee (normalizing practices). Hence, both Indian high-biodiverse and high shade-grown coffee farming and more sun-exposed coffee farms in Brazil and Vietnam fulfill RA and UTZ biodiversity criteria and are sold under the same certification label (Rainforest Alliance 2014; UTZ Certified 2015).

The links between representational/normalizing practices and exchange practices (links B and C in Fig. 5) further strengthen the configurational fit between mainstream coffee market practices. Exchange of biodiverse coffee on the mainstream market builds on large supplies of certified coffee across countries and management systems, which require acceptance of VSS certification as a legitimate biodiversity indicator. Hence in Kodagu recruitment and group certification of coffee farms as well as preferred supplier schemes rest on VSS standards that do not discriminate between of high and low biodiversity in coffee farming (link B direction representational to exchange practices and link C direction normalizing to exchange practices). Exchange practices are translated into representational and normalizing practices (link B direction exchange to representational practices and link C direction exchange to normalizing practices). By demanding certification, creating a long-term supplier base with certified coffee farmers, and communicating the distinct sustainability qualities of VSS labeled coffee, large international market actors sustain images and perceptions of VSS as a guarantee for biodiversity conservation in coffee farming. VSS biodiversity standards and criteria simultaneously frame and perform mainstream coffee market exchange.

### The Configurational Fit between Mainstream Coffee Market Practices and Outcomes for the Market Value of High-Biodiversity Coffee

The reinforcing capacity of translations between exchange, representational and normalizing mainstream coffee market practices attributed to RA and UTZ certification produce specific outcomes for the marketing of high-biodiverse coffee. These links have a reinforcing capacity, affecting one another. The configurational fit between mainstream coffee market practices suggests that outcomes for the marketing potential of high-biodiversity, shade-grown coffee might be severe. Our results clearly indicate that the size of financial remuneration, in terms of very small price premiums for mass market-certified coffee, potentially could jeopardize the marketing value of Indian shade-grown high-biodiversity coffee. The pressure to increase coffee income through short-term productivity gains by opening of shade and replacing native trees with commercial ones, affects biodiversity in terms of species diversity and richness. In Kodagu high-biodiverse setting, it is possible for coffee farmers to increase coffee yields by decreasing shade (and thus biodiversity) and at the same time fulfill RA and UTZ biodiversity standards. However, the opening of shade affects the inherent and unique flavor of high-biodiversity, shade-grown coffee that is the main benefit on which the marketing of high-biodiversity coffee can capitalize (Upendranadh and Subbaiah 2012; Vaast et al. 2006). Hence due to pressures to increase coffee income on the market for mainstream coffee, the very basis for marketing high-biodiversity, shade-grown coffee is threatened. The unique value of high-biodiversity, shade-grown coffee is seemingly incompatible with a mainstream coffee market with a focus on quantity of sustainable coffee. Mainstream coffee market VSS certification schemes as the RA and UTZ serve as focal actors safeguarding a focus on quantity, not quality, of biodiverse coffee exchange. Thus, the contribution of mainstream coffee market VSS certifications to the configurational fit of this market is very strong. Even though approximately half of the certified Kodagu coffee is sold as non-certified at farm gates, which suggests that there exist a considerable overflow in relation to the framing qualities assigned to certification, certification affects the marketing value (associated with taste) of this coffee.

## Conclusions

By adopting a performative perspective, this article illustrates how VSS biodiversity criteria take part in mainstream coffee market configuration. Outcomes of VSS biodiversity criteria for diversification of high-biodiversity, shade-grown coffee are found on two levels. First, these criteria produce coffee farming in alignment with criteria specifications. Second, alignment with biodiversity criteria will affect the unique flavor of this coffee. Thus, our analysis clearly shows that high-biodiversity and shade-grown coffee cannot make it on the mainstream coffee market. Mutually reinforcing market perceptions as well as financial remuneration and supply schemes in the mainstream coffee market are incompatible with high-biodiversity coffee-farming practices, which provide the core benefit of this coffee, namely, its unique flavor. Hence, there is little potential for marketing efforts to diversify coffee based on biodiversity conservation in coffee farming within this market.

This article provides an understanding for how marketing initiatives (including VSS certification) shape the market for high-biodiversity and shade-grown coffee. Whereas earlier studies have investigated what economic benefits to farmers and what biodiversity criteria connected to coffee certification schemes that affect high-biodiversity coffee farming (Gobbi 2000; Mas and Dietsch 2004; Perfecto et al. 2005; Philpott et al. 2007), we show that coffee marketing and branding tools are performative and produce different biodiversity outcomes. Our results expand the understanding of how markets are shaped by VSS certification. We show how VSS as a marketing device shape the mainstream coffee market through mutual reinforcing market practices as large-scale supply of low-priced sustainable coffee and low standards for biodiversity conservation in coffee farming. For coffee, a product for which the environmental properties of production are intimately connected to recognizable properties such as flavor, diversification marketing strategies are dependent on the support of dominant coffee roasters through incentives for biodiversity conservation in coffee farming.

### Managerial Implications

The contribution of RA and UTZ certifications to the configurational fit between market practices in the mainstream market has implications for a discussion about alternative approaches to marketing high-biodiversity, shade-grown coffee in this market. The literature on coffee farming and biodiversity conservation primarily discusses two initiatives that have the potential to provide economic incentives for biodiversity conservation through coffee diversification: shade coffee certification and geographical indications (GIs) of origin (Perfecto et al. 2005; Teuber 2010; Upendranadh and Subbaiah 2012).

The probability that shade coffee certification as a focal actor can script the mainstream coffee market through influencing coffee market actors’ perceptions of biodiversity in coffee farming, and subsequently their business models, depends on the relative power of such certification in terms of access to resources, of information and relationships, and of skills (Fligstein 2001; Storbacka and Nenonen 2011b; Zaheer and Bell 2005). The success of such programs depends on the willingness of coffee consumers and coffee roasters to pay price premiums for high-biodiversity coffee (Giovannucci and Koekoek 2003; Perfecto et al. 2005). It seems that the current dominant business models in the mainstream coffee market, with its heavy reliance on high-quantity, anonymous, and not premium-priced biodiversity coffee as an integral part of sustainable coffee branding, will not be easily changed based on higher prices.

Another stream of coffee marketing literature discusses GIs of origin as a means of coffee diversification that has the potential to provide economic benefits for high-biodiversity coffee. GIs are defined as “indications, which identify a good as originating in the territory of a Member (of the WTO, author remark), or a region or locally in that territory, in which a given quality, reputation or other characteristics of the good [is] essentially attributable to its geographical origin” by The Agreement on Trade-Related Aspects of Intellectual Property Rights (WTO 1994). The marketing potential of single-origin coffees compares with the diversification and value creation of fine wines (Daviron and Ponte 2005; Teuber 2010). GI protection is undertaken at the country level or at a regional level (see Teuber 2010 for a detailed account). The EU distinguishes between two certifications for GIs: protected designations of origin (PDOs), which require all stages of coffee production to occur in the geographical area in question, and protected geographical indications (PGIs), which require that a minimum of one stage of coffee production is located in the specific area (Teuber 2010). Coffee will become PGIs rather than PDOs because roasting in most cases occurs outside the area or country of origin. Café de Columbia is the most well-known coffee PGI to date. Trademarks are another means of protecting GIs. “A trademark is a word, phrase, symbol, and/or design that identifies and distinguishes the source of the goods of one party from those of others” (USPTO 2015). The government of Ethiopia considers this the better option for protecting coffee GIs, and Harrar, Sidamo, and Yirgacheffe are registered trademarks in the EU and in the United States (Teuber 2010). However, coffee trademarks differ from coffee PGI certification on one important measure, that is, trademarks do not assure any links to quality comparable to how PGIs establish a connection between certain characteristics and origin (Teuber 2010).

The scripting propensity of coffee GIs in the mainstream coffee market is most likely stronger than that of the above-discussed shade-coffee certifications. Coffee PGIs and coffee trademarks will indirectly contribute to high-biodiversity, shade-grown coffee farming if they are successful in establishing a connection between coffee taste valued/in demand by coffee consumers and coffee origin, and if the value of this coffee is transferred to a coffee farmer(s). However, such scripting strength is dependent on marketing resources and outreach on the global coffee market (Storbacka and Nenonen 2011a, b; Zaheer and Bell 2005), on which roasters buy coffee with detailed information about quality but release very little of this information to coffee consumers (Ponte and Gibbon 2005). Without a financial incentive, current business models, with their heavy reliance on high-quantity and anonymous coffee as an integral part of sustainable coffee branding, will not be easily changed. Coffee trademarks can be licensed to international roasters and thus act as leverage for single-origin coffee in mass market brand building. Additionally, PGIs can be part of government support of estate branding and schemes to boost domestic coffee demand in coffee-producing countries (Upendranadh and Subbaiah 2012).
